# Supplementary material for: OTUD5 cooperates with TRIM25 in transcriptional regulation and tumor progression via deubiquitination activity
Source: Nat Commun. 2020 Aug 21;11:4184. doi: 10.1038/s41467-020-17926-7 (PMC7442798; doi:10.1038/s41467-020-17926-7)
Supplement: Supplementary file 1 — Supplementary Information [file 41467_2020_17926_MOESM1_ESM.pdf]

Supplementary Information

OTUD5 cooperates with TRIM25 in transcriptional regulation and tumor progression via deubiquitination activity

Fangzhou Li et al.

Supplementary Figure 1.

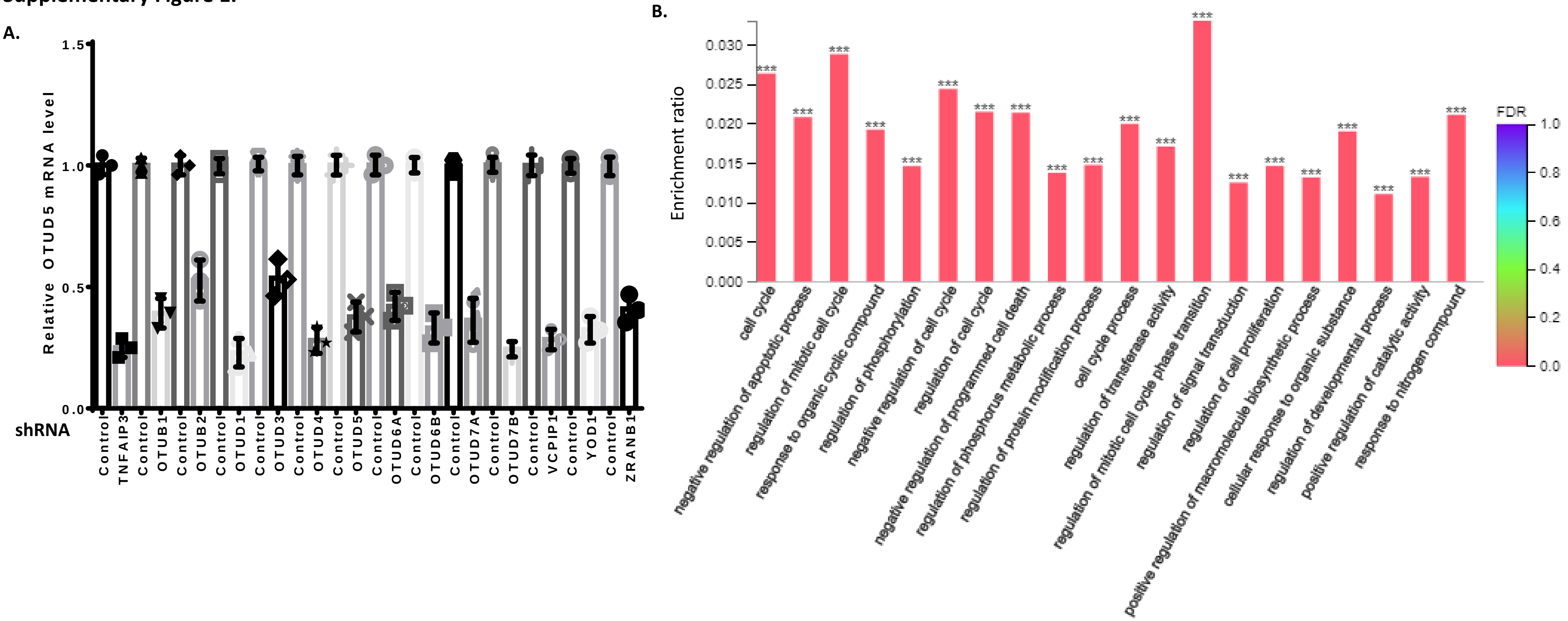

Supplementary figure 1. (A) Knockdown efficiency of the OTU DUBs was verified by RT-PCR analysis. Data are represented as means ± SD from three biological experiments. **(B)** Gene ontology (GO) analyses of the differentially expressed genes in the TRIM25 depleted cells, n = 3 (siControl, siTRIM25-1 or siRNA-2) biological independent samples. ****p* < 0.001. Source data are provided as a Source Data file.

Supplementary Figure 2.

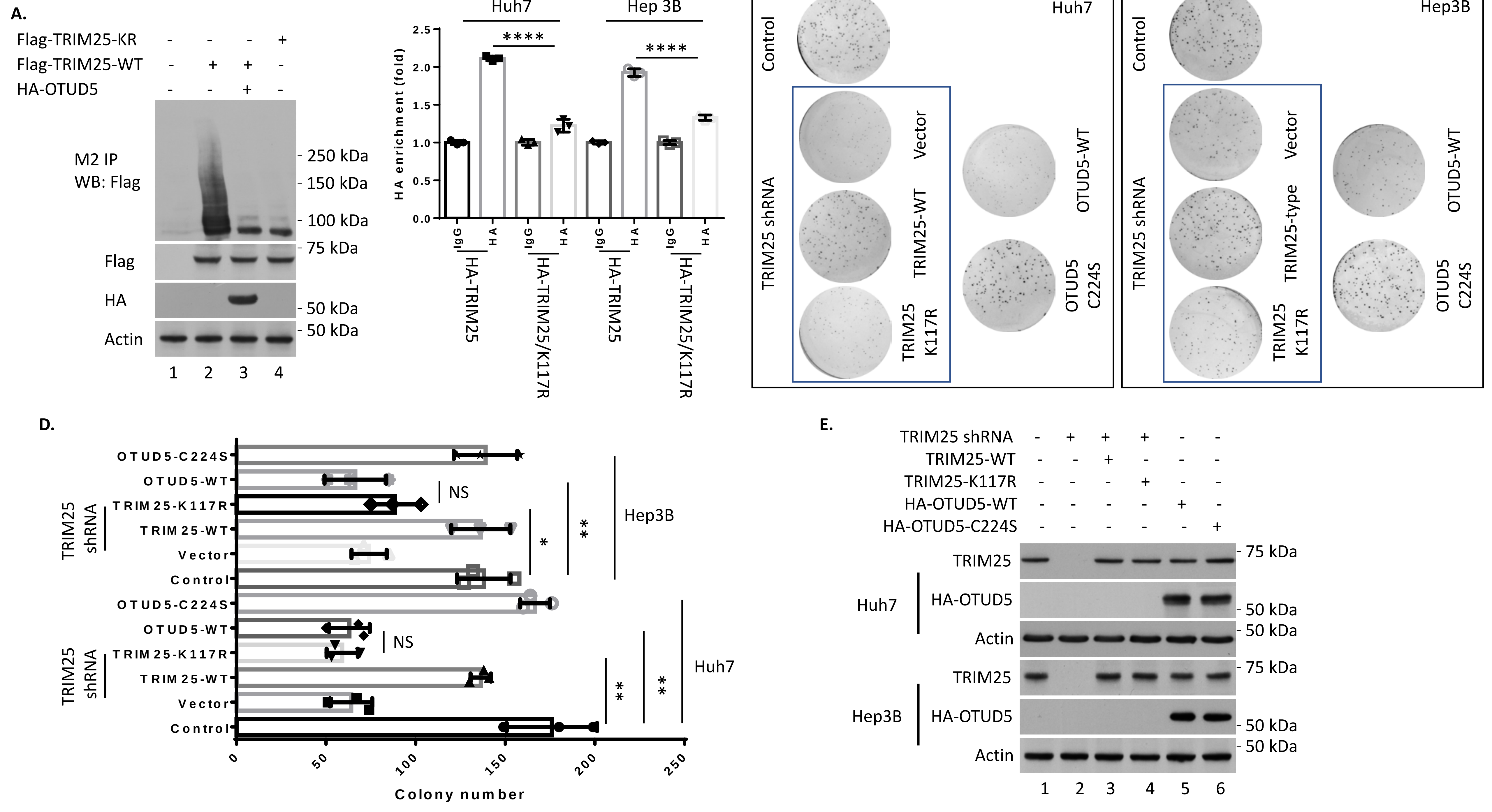

Supplementary figure 2. (A) K117 was the key residue for TRIM25 autoubiquitination. 293T cells were co-transfected with Flag-TRIM25-wild type (WT) and vector plasmids (lane 2) or plasmids encoding HA-OTUD5 (lane 3) or Flag-TRIM25-K117R (KR) (lane 4). **(B)** Huh7 and Hep3B cells were transfected with plasmids expressing HA-TRIM25 or HA-TRIM25/K117R, and then subjected to ChIP analysis with anti-HA antibody. RT-PCR with ChIP samples was carried out, and the results are plotted as indicated. Fold enrichment indicates the recruitment of HA-TRIM25 compared with that of an IgG control. Data show the mean of three biological independent experiments. Data are represented as means ± SD from three biological experiments. ****$p < 0.0001$ (TRIM25 versus TRIM25/K117R in the Huh7 cells), ****$p < 0.0001$ (TRIM25 versus TRIM25/ K117R in the Hep3B cells), two-tailed unpaired t-test. **(C-D)** The effect of the TRIM25 K117R mutant and OTUD5 on cell proliferation was determined by colony formation assay. **(E)** Western blot analysis was performed to confirm the expression level of TRIM25 or HA-OTUD5 in the cells used for C and D. Data are represented as means ± SD from three biological experiments. **$p = 0.0016$ (Control versus TRIM25/K117R in the Huh7 cells), **$p = 0.0021$ (Control versus OTUD5 in the Huh7 cells), *$p = 0.0137$ (Control versus TRIM25/K117R in the Hep3B cells), **$p = 0.0055$ (Control versus OTUD5 in the Hep3B cells), two-tailed unpaired t-test. NS, non-significance. Source data are provided as a Source Data file.

Supplementary Figure 3.

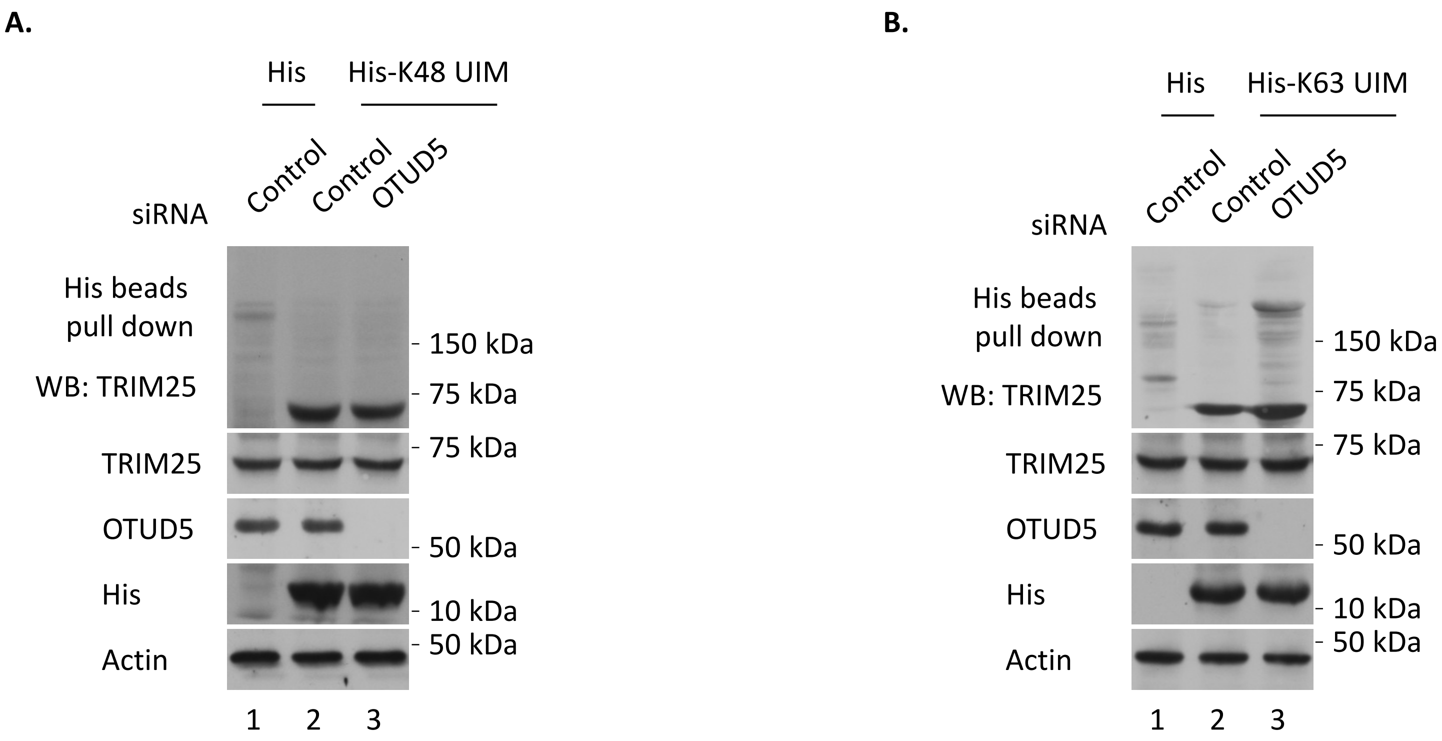

Supplementary figure 3. OTUD5 knockdown increased the K63 ubiquitination of TRIM25 in Hep3B cells. (A) His beads (lane 1) or His-K48-UIM fusion beads (lanes 2 and 3) were incubated with the cell extracts of Hep3B cells transfected with control siRNA (lanes 1 and 2) or siRNA targeting OTUD5 (lane 3). The IP-ed proteins and cell lysates were subjected to Western blot analysis using antibodies against TRIM25, OTUD5, His and Actin. **(B)** Wild-type (lane 1 and lane 2) or OTUD5 depleted (lane 3) Hep3B cells were subjected to His beads IP (lane 1) or His-K63-UIM fusion beads IP (lane 2 and lane 3). Hep3B cells were transfected with control siRNA or siRNA targeting OTUD5, and after 48 hours, IP was conducted. The eluates of IP and cell lysates were analysed by Western blotting using the indicated antibodies. Source data are provided as a Source Data file.

Supplementary Figure 4.

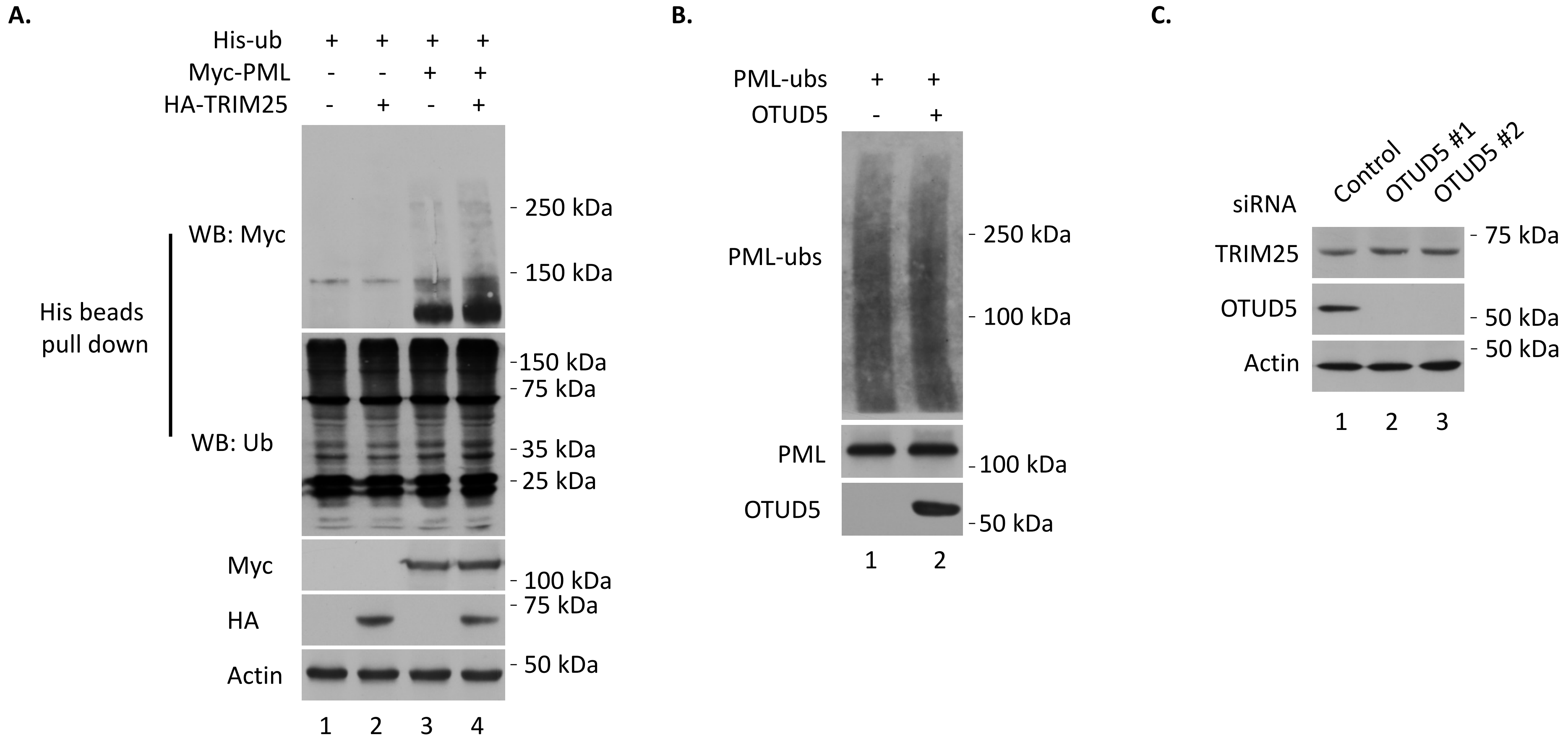

Supplementary figure 4 (A) TRIM25 promoted PML ubiquitination. 293T cells were transiently transfected with expression constructs encoding Myc-PML, HA-TRIM25, and His-ub. The eluates of His pull down and cell lysates were analysed by Western blotting using the indicated antibodies. **(B)** OTUD5 cannot deubiquitinate PML *in vitro*. OTUD5 was incubated with PML-ubs for *in vitro* assay. **(C)** Hep3B cells were transfected with one of the two siRNAs (#1, #2) (lanes 2 - 3) or control siRNA (lane 1) for 48 hours. The chromatin fraction was extracted by chromatin extract buffer (20 mM HEPES pH 7.9, 50 kDa mM NaCl, 300 mM sucrose, 3 mM $MgCl_2$, and 0.5% Triton X-100) [66]. The lysate was analysed by Western blotting using the indicated antibodies. Source data are provided as a Source Data file.

Supplementary Figure 5.

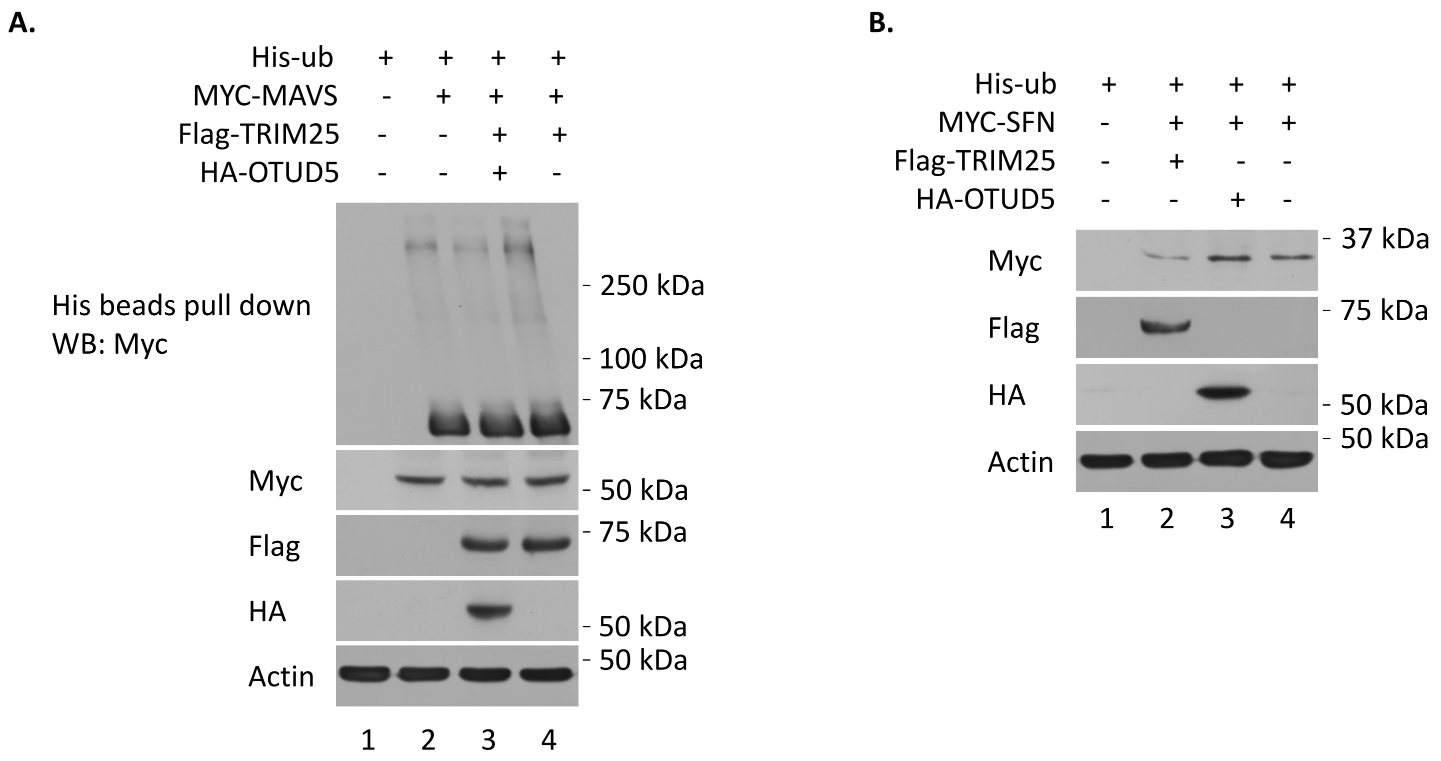

Supplementary figure 5 OTUD5 played a role in ubiquitination of TRIM25 targets. (A) OTUD5 reduced the TRIM25-induced ubiquitination level of MAVS. Ubiquitinated MAVS was pulled down from 293T cells transiently transfected with expression constructs encoding Myc-MAVS, Flag-TRIM25, and His-ub with or without HA-OTUD5. The products from the f Ni-NTA pull-down assay were analysed by Western blot using an antibody against MYC-tag. The input was subjected to Western blot analysis using antibodies against Myc, HA, Flag and Actin. **(B)** OTUD5 maintains the stability of SFN. 293T cells were transiently transfected with expression constructs encoding Myc-SFN, Flag-TRIM25 and HA-OTUD5 as indicated. The cell lysates were analysed by Western blotting using antibodies against Myc, HA, Flag and Actin. Source data are provided as a Source Data file.

Supplementary Figure 6.

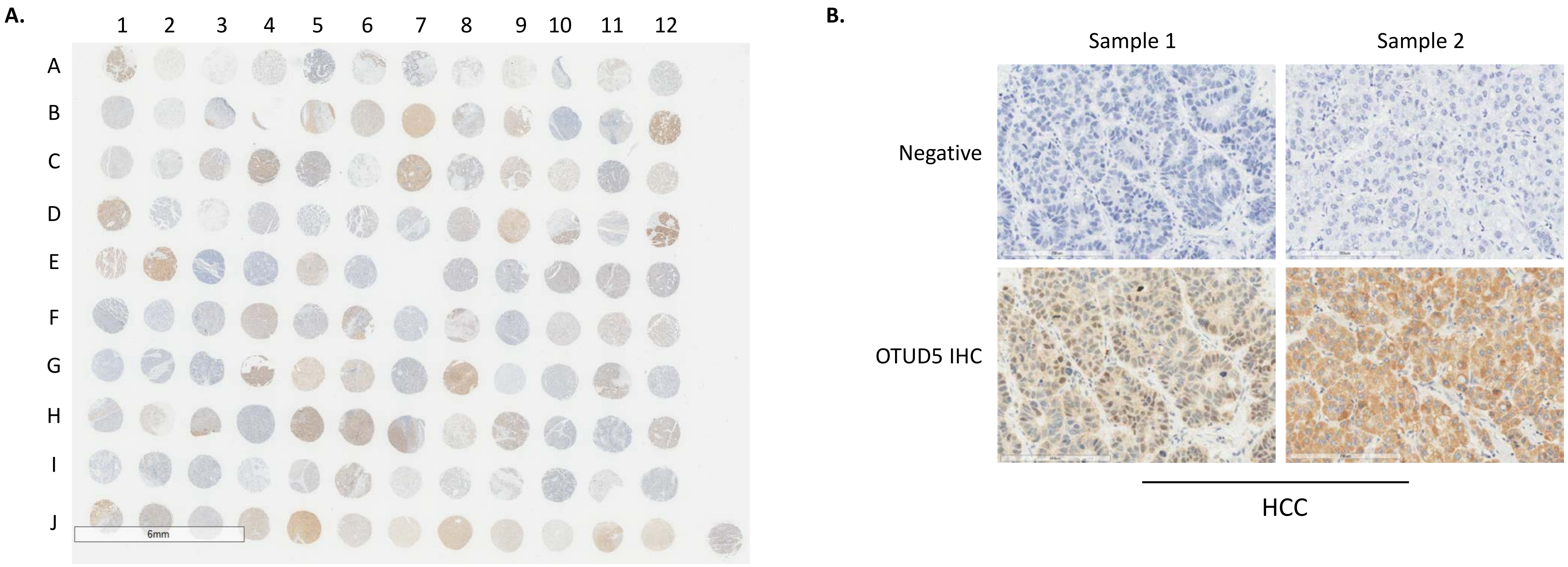

Supplementary figure 6. (A-B) Specificity and cellular localization of OTUD5 were determined for the HCC tissue from cohort 1 patients. HCC tissues were stained with anti-OTUD5 antibody. (A) Scale bar, 6 mm. (B) Scale bar, 200 um.

Supplementary Figure 7.

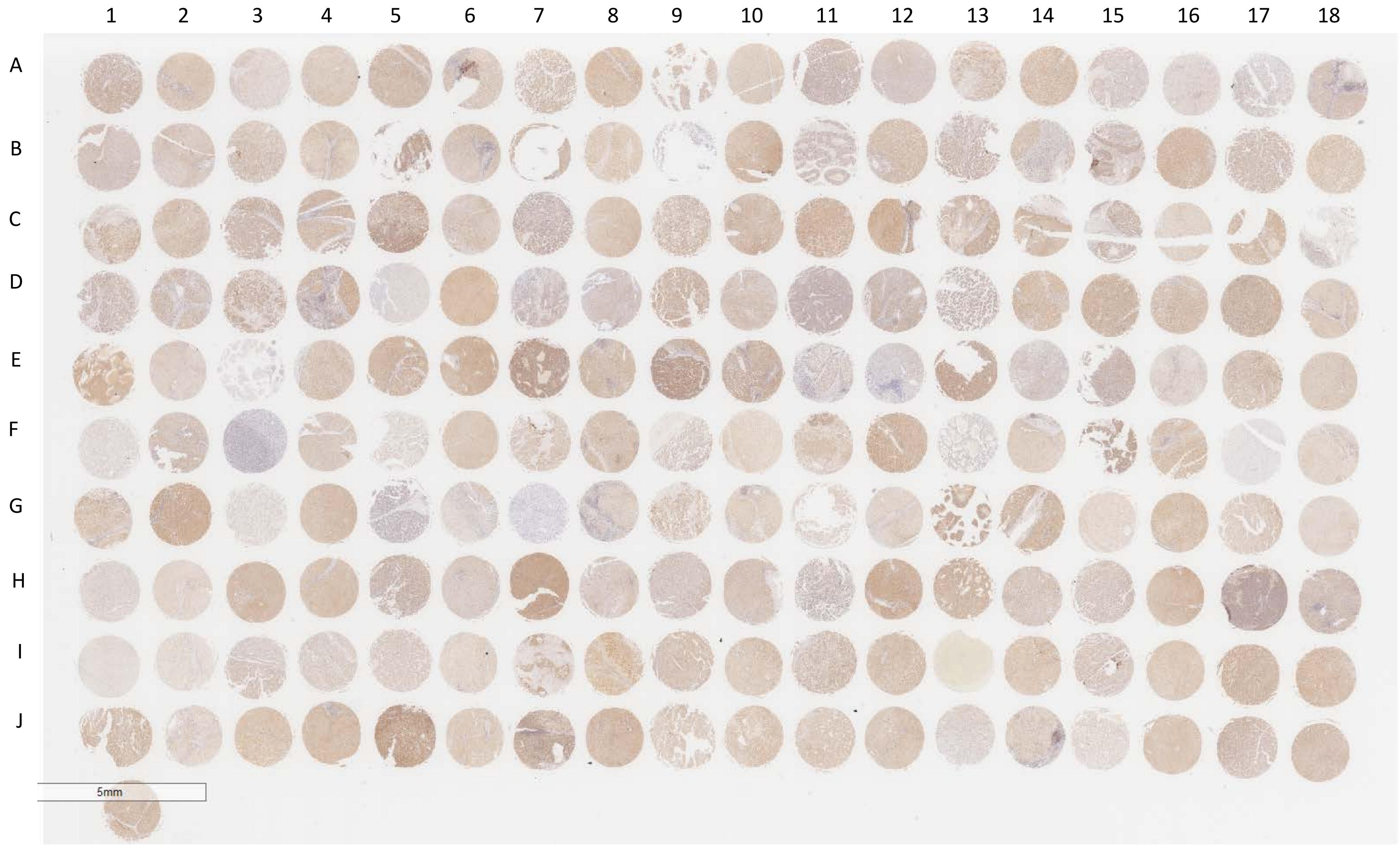

Supplementary figure 7. Specificity and cellular localization of OTUD5 were determined for the HCC tissue from the cohort 2 patients. A total of 90 of paired noncancerous and tumor tissues from the HCC patients were stained with anti-OTUD5 antibody. Scale bar, 5 mm.
