# Supplementary material for: OTUD5 cooperates with TRIM25 in transcriptional regulation and tumor progression via deubiquitination activity
Source: Nat Commun. 2020 Aug 21;11:4184. doi: 10.1038/s41467-020-17926-7 (PMC7442798; doi:10.1038/s41467-020-17926-7)
Supplement: Supplementary file 3 — Description of Additional Supplementary Files [file 41467_2020_17926_MOESM3_ESM.pdf]

Description of Additional Supplementary Files

File Name: Supplementary Data 1
Description: Contains the results of OTUD5 complex Mass Spectrometry.

File Name: Supplementary Data 2
Description: Contains the sequences of siRNA and shRNA.

File Name: Supplementary Data 3
Description: Contains the results of RNA sequencing.

File Name: Supplementary Data 4
Description: Contains the List of primers used in the study.

File Name: Supplementary Data 5
Description: Contains the results of OTUD5 IHC staining in microarray from tissues of normal liver and primary hepatic cancer.

File Name: Supplementary Data 6
Description: Contains the results of OTUD5 IHC staining in microarray from HCC and the paired non-tumorous tissues.

File Name: Supplementary Data 7
Description: Contains the demographical and clinical features of NSCLC patients in this study.
