# Supplementary material for: OTUD5 cooperates with TRIM25 in transcriptional regulation and tumor progression via deubiquitination activity
Source: Nat Commun. 2020 Aug 21;11:4184. doi: 10.1038/s41467-020-17926-7 (PMC7442798; doi:10.1038/s41467-020-17926-7)
Supplement: Supplementary file 11 — Reporting Summary [file 41467_2020_17926_MOESM11_ESM.pdf]

nature research

Corresponding author(s): Wenhui Zhao; Dawei Li

Last updated by author(s): Jul 9, 2020

Reporting Summary

Nature Research wishes to improve the reproducibility of the work that we publish. This form provides structure for consistency and transparency in reporting. For further information on Nature Research policies, see Authors & Referees and the Editorial Policy Checklist.

Statistics

For all statistical analyses, confirm that the following items are present in the figure legend, table legend, main text, or Methods section.

n/a	Confirmed	
☐	☒	The exact sample size (*n*) for each experimental group/condition, given as a discrete number and unit of measurement
☐	☒	A statement on whether measurements were taken from distinct samples or whether the same sample was measured repeatedly
☐	☒	The statistical test(s) used AND whether they are one- or two-sided<br>*Only common tests should be described solely by name; describe more complex techniques in the Methods section.*
☒	☐	A description of all covariates tested
☒	☐	A description of any assumptions or corrections, such as tests of normality and adjustment for multiple comparisons
☐	☒	A full description of the statistical parameters including central tendency (e.g. means) or other basic estimates (e.g. regression coefficient) AND variation (e.g. standard deviation) or associated estimates of uncertainty (e.g. confidence intervals)
☐	☒	For null hypothesis testing, the test statistic (e.g. *F*, *t*, *r*) with confidence intervals, effect sizes, degrees of freedom and *P* value noted<br>*Give P values as exact values whenever suitable.*
☒	☐	For Bayesian analysis, information on the choice of priors and Markov chain Monte Carlo settings
☐	☒	For hierarchical and complex designs, identification of the appropriate level for tests and full reporting of outcomes
☐	☒	Estimates of effect sizes (e.g. Cohen's *d*, Pearson's *r*), indicating how they were calculated

Our web collection on statistics for biologists contains articles on many of the points above.

Software and code

Policy information about availability of computer code

Data collection	BD bioscience FACSCalibur was used to collect flow cytometric data. QuantStudio Dx Instrument (Applied Biosystems) was used to collect Real-time PCR data.
Data analysis	The data analysis was performed by Image Lab software (Version 3.0 beta), BD FACStation software version 6.0, Microsoft Excel (Version 11.0) and GraphPad Instat software (Version 5.01).

For manuscripts utilizing custom algorithms or software that are central to the research but not yet described in published literature, software must be made available to editors/reviewers. We strongly encourage code deposition in a community repository (e.g. GitHub). See the Nature Research guidelines for submitting code & software for further information.

Data

Policy information about availability of data

All manuscripts must include a data availability statement. This statement should provide the following information, where applicable:

- Accession codes, unique identifiers, or web links for publicly available datasets
- A list of figures that have associated raw data
- A description of any restrictions on data availability

Data availability
Statistical source data for the graphical representations and statistical analyses in figure 1–8 and Supplementary figs. 1–5 are provided as a Source Data file. Source data are provided with this paper. The mass spectrometry proteomics data are available via ProteomeXchange with identifier PXD019907. The RNA-seq data could be accessible with the following link after the indicated release date: https://www.ncbi.nlm.nih.gov/sra/PRJNA640513. The publicly available mRNA expression and clinical information (TCGA database) were downloaded from the cBioPortal for Cancer Genomics (http://www.cbioportal.org/). All the raw data and materials in this manuscript are available upon request.

Field-specific reporting

Please select the one below that is the best fit for your research. If you are not sure, read the appropriate sections before making your selection.

☒ Life sciences ☐ Behavioural & social sciences ☐ Ecological, evolutionary & environmental sciences

For a reference copy of the document with all sections, see nature.com/documents/nr-reporting-summary-flat.pdf

Life sciences study design

All studies must disclose on these points even when the disclosure is negative.

Sample size	All sample size was revealed in the sections of Materials and Methods or Figure Legends. For no sample size calculation was performed, we have chosen several large cohorts to perform statistical analysis, the sample size of which is sufficient for correlation analysis, Chi-square or Fisher's test. We also used publicly available TCGA database from large cohorts of tumor patients to perform survival analysis. For student's t test, at least 3 biological repeats was used to calculate p value. The sample size is commonly acceptable in biological experiments.
Data exclusions	A total of 110 tumor samples were immunostained in the tissue microarray for primary liver cancer. The staining for E7 was missing. The staining for J1 was excluded from the statistics because of mixed hepatocellular carcinoma and normal live tissue. For OTUD5 expression correlation with demographic and clinical features in LUAD patients, the cutoff value for OTUD5 mRNA expression is set at 10 and two patients were excluded from the analysis. All the inclusion and exclusion criteria were stated in details in the main text. These exclusion criteria were pre-established because the expression level of OTUD5 in 2 patients out of 522 LUAD patients was extremely and abnormally higher in comparison with the others. We considered that the experimental or sample issues affect the data accuracy and therefore excluded the data of 2 patients from the final statistical analysis.
Replication	Each experiment was performed in three biological replicates with consistent results.
Randomization	The research subjects were randomly assigned to the treatment or control group. Nude mice were chosen as xenograft models, and randomly allocated into experimental groups.
Blinding	For in vitro cell-based experiments, the investigators were not blinded during data acquisition and analysis. The application of treatments and processing procedures made it difficult for blinding but there was no human bias given all the data were collected independently using instrumentation. For the animal experiments the investigators were not blinded to the group allocation. For in vitro cell-based experiments, the investigators were not blinded during data acquisition and analysis. The application of treatments and processing procedures made it difficult for blinding but there was no human bias given all the data were collected independently using instrumentation. For the animal experiments the investigators were not blinded to the group allocation. During this process for cell and mouse experiments, only one researcher performed an experiment at a time and therefore it is difficult to blind him to the experiment. However, we ensure that the outcomes being measured are as objective as possible. We used duplicate assessment of outcomes and reported the level of agreement achieved by the investigators. At least two observers measured xenograft tumor volumes/weights or the latency of tumor formation/survival time to alleviate human bias in these data. All immunohistochemistry sections were blindly assessed by two pathologists independently. The third pathologist's assessment is performed if controversial results are acquired.

Reporting for specific materials, systems and methods

We require information from authors about some types of materials, experimental systems and methods used in many studies. Here, indicate whether each material, system or method listed is relevant to your study. If you are not sure if a list item applies to your research, read the appropriate section before selecting a response.

Materials & experimental systems

n/a	Involved in the study	
☐	☒	Antibodies
☐	☒	Eukaryotic cell lines
☒	☐	Palaeontology
☐	☒	Animals and other organisms
☐	☒	Human research participants
☒	☐	Clinical data

Methods

n/a	Involved in the study	
☒	☐	ChIP-seq
☐	☒	Flow cytometry
☒	☐	MRI-based neuroimaging

Antibodies

Antibodies used	The following primary antibodies were used at a dilution of 1:1000: anti-OTUD5 (D8Y2U, Cell Signaling Technology), anti-TRIM25 (Abcam, ab167154), anti-PML (Abcam, ab53773), anti-HA (H9658, Sigma), anti-Flag (F3165, Sigma) and anti-ubiquitin (SC-8017, Santa Cruz). Anti-β-actin (Sigma, AC-15) primary antibody was used at a dilution of 1:5000. Goat anti-Rabbit IgG Secondary Antibody HRP conjugated (SAB Signalway Antibody, L3012) and Goat anti-Mouse IgG Secondary Antibody HRP conjugated (SAB Signalway Antibody, L3032) were used at a dilution of 1:5000.

Validation	Antibodies critical for novel conclusions were validated by elimination of signals upon knock-down experiments and/or by functional assays. All antibodies were used in the system under study (assay and species) according to the profile of manufacturer.

Eukaryotic cell lines

Policy information about cell lines

Cell line source(s)	Hep3B, Huh7 and 293T cells were purchased from National Infrastructure of cell line resource (Beijing, China).
Authentication	Routine fingerprinting and mycoplasma testing was performed.
Mycoplasma contamination	All the cell lines were obtained from National Infrastructure of cell line resource and have been proven to be negative for mycoplasma contamination.
Commonly misidentified lines (See ICLAC register)	No cell lines used in this work were listed in the ICLAC database.

Animals and other organisms

Policy information about studies involving animals; ARRIVE guidelines recommended for reporting animal research

Laboratory animals	4- to 6-week-old male nude mice (NU/NU; 4-6 weeks old; male; strain 403; Charles River) were licensed by Beijing Municipal Committee of Science and Technology. All mice were housed in a temperature-controlled room (22 ± 2 °C) with 40-60% humidity, with a light/dark cycle of 12h/12h. All animal experiments were done according animal protocol (No. LA2018013) to Wenhui Zhao approved by the Animal Ethics Committee of the Peking University Health Science Center, China.
Wild animals	No wild animals were used in this study.
Field-collected samples	No field-collected samples were used in this study.
Ethics oversight	The study is compliant with all relevant ethical regulations for animal experiments. All the experimental protocols were approved by the Institutional Animal Care and Use Committee of Peking University.

Note that full information on the approval of the study protocol must also be provided in the manuscript.

Human research participants

Policy information about studies involving human research participants

Population characteristics	The demographical and clinical features of all liver cancer and NSCLC patients are shown in supplementary Table 3 and 4.
Recruitment	We also assessed OTUD5 expression in 21 non-small-cell lung carcinoma (NSCLC) patients. The patients who underwent curative surgery at The Affiliated Zhangjiagang Hospital of Soochow University from March 2019 to July 2019 were enrolled in this study. Histological confirmation of primary NSCLC was obtained from the Department of Pathology at the hospital. None of the patients received preoperative adjuvant chemotherapy.<br><br>We cannot recruit the NSCLC patients with late-stage diseases in this study, especially for those patients with distant metastasis, who are not fit for curative surgery. Therefore, most of the patients included in the current study were from early-stage NSCLC patients. Further study was required to determine OTUD5 expression and its correlation with PML level in more progressive NSCLC patients.
Ethics oversight	The written permission was requested and received from all NSCLC patients in the study. The use of the human specimens was approved by The Zhangjiagang Hospital Institutional Review Board (No.2019001). To determine OTUD5 expression in primary liver cancer, we purchased commercial available human liver tissue microarray (ALENA biotechnology; Xi'an; China).

Note that full information on the approval of the study protocol must also be provided in the manuscript.

Flow Cytometry

Plots

Confirm that:

☒ The axis labels state the marker and fluorochrome used (e.g. CD4-FITC).

☒ The axis scales are clearly visible. Include numbers along axes only for bottom left plot of group (a 'group' is an analysis of identical markers).

☒ All plots are contour plots with outliers or pseudocolor plots.

☒ A numerical value for number of cells or percentage (with statistics) is provided.

Methodology

Sample preparation	The cells were plated at 50-70% of confluency with 500 nM TSA dissolved in DMSO or not. After 24 hours, the cells were collected and stained with propidium iodide (PI) for flow cytometric analysis.
Instrument	FACSCalibur, BD bioscience
Software	FACADiva software
Cell population abundance	10,000 cells were analyzed for each sample
Gating strategy	Staining is with a nucleic acid dye like PI and is analyzed according to the standard procedure.

☒ Tick this box to confirm that a figure exemplifying the gating strategy is provided in the Supplementary Information.
